# Supplementary material for: Complexome profiling of the Chlamydomonas psb28 mutant reveals TEF5 as an early PSII assembly factor
Source: Plant Cell. 2025 Mar 17;37(6):koaf055. doi: 10.1093/plcell/koaf055 (PMC12164590; doi:10.1093/plcell/koaf055)
Supplement: koaf055_Supplementary_Data [file koaf055_supplementary_data.zip › psb28_interactive.zip!/plots/intensity/qualityPlots/peptides/DII4_Cre13.g603700.t1.2.html]

### Cre13.g603700.t1.2

Trivial name: DII4  
SeqCoverage [median]: 25.20  
Mass: 41.84 kDa  
  
 

| Sequence | MissedCleavage | Mass [Da] | wtIntensity | muIntensity | Score |
| --- | --- | --- | --- | --- | --- |
| AGFAGDDAPR | 0 | 975.44 | 3433.16 | 492.88 | 122.13 |
| AVFPSIVGR | 0 | 944.54 | 11238.57 | 0.00 | 116.90 |
| AVFPSIVGRPR | 1 | 1197.70 | 104649.70 | 0.00 | 143.89 |
| DLTDYLMK | 0 | 997.48 | 3888.41 | 0.00 | 151.56 |
| DSYVGDEAQSK | 0 | 1197.52 | 43476.45 | 0.00 | 219.72 |
| DSYVGDEAQSKR | 1 | 1353.62 | 82227.17 | 0.00 | 171.49 |
| EITALAPSAMK | 0 | 1130.60 | 913.97 | 721.97 | 100.02 |
| EIVRDIKEK | 2 | 1128.65 | 4538.27 | 0.00 | 100.48 |
| GYSFTTTAER | 0 | 1131.52 | 45181.32 | 0.00 | 182.98 |
| GYSFTTTAEREIVR | 1 | 1628.82 | 15868.07 | 0.00 | 126.09 |
| LDLAGRDLTDYLMK | 1 | 1622.83 | 29882.46 | 805.43 | 112.24 |
| VAPEEHPVLLTEAPLNPK | 0 | 1953.06 | 89344.88 | 1111.18 | 201.75 |
|  |  |  | 434642.4 | 3131.5 |  |
